# Marmoset core visual object recognition behavior is comparable to that of macaques and humans

**DOI:** 10.1016/j.isci.2022.105788

**Published:** 2022-12-10

**Authors:** Alexander J.E. Kell, Sophie L. Bokor, You-Nah Jeon, Tahereh Toosi, Elias B. Issa

**Affiliations:** 1 Zuckerman Mind Brain Behavior Institute, Columbia University, New York, NY 10027, USA; 2 Department of Neuroscience, Columbia University, New York, NY 10027, USA

**Keywords:** Biological sciences, Neuroscience, Sensory neuroscience

## Abstract

Among the smallest simian primates, the common marmoset offers promise as an experimentally tractable primate model for neuroscience with translational potential to humans. However, given its exceedingly small brain and body, the gap in perceptual and cognitive abilities between marmosets and humans requires study. Here, we performed a comparison of marmoset behavior to that of three other species in the domain of high-level vision. We first found that marmosets outperformed rats – a marmoset-sized rodent – on a simple recognition task, with marmosets robustly recognizing objects across views. On a more challenging invariant object recognition task used previously in humans, marmosets also achieved high performance. Notably, across hundreds of images, marmosets’ image-by-image behavior was highly similar to that of humans – nearly as human-like as macaque behavior. Thus, core aspects of visual perception are conserved across monkeys and humans, and marmosets present salient behavioral advantages over other small model organisms for visual neuroscience.

## Highlights

• We compared object recognition in four species spanning the evolution of mammalian vision
• Marmosets far outperformed rodents in object recognition
• Marmoset object recognition was similar in pattern to macaques and humans
• Similar in size to rats, marmosets provide an alternative for visual neuroscience

## Graphical abstract

**Figure:**
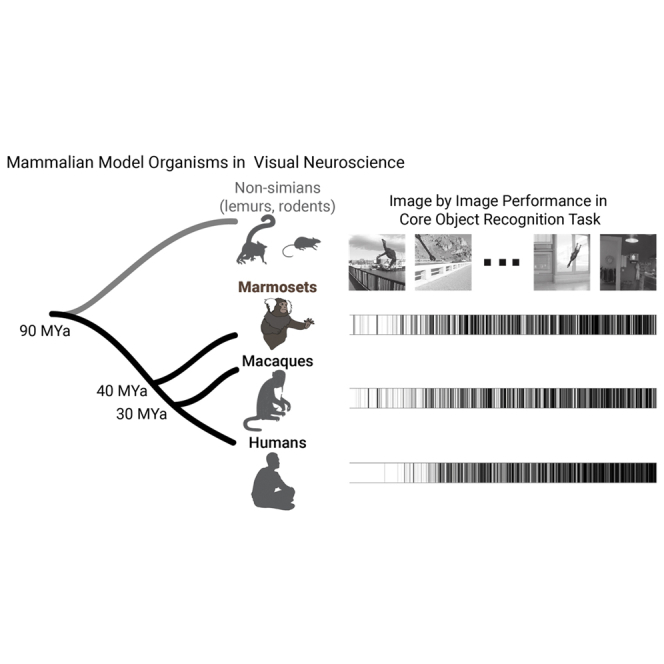

## Introduction

Descended from a common ancestor, the simian primates consist of monkeys, apes, and humans. Among the smallest of the simians, the common marmoset (*Callithrix jacchus*) was introduced to neuroscience decades ago^1^^,^^2^^,^^3^^,^^4^ and has recently been adopted more broadly.^5^^,^^6^^,^^7^^,^^8^^,^^9^^,^^10^^,^^11^^,^^12^^,^^13^^,^^14^^,^^15^ In contrast to larger simian primate model organisms like rhesus macaques, marmosets may mitigate traditional compromises between an animal model’s methodological tractability and its phylogenetic proximity to humans, offering features typically associated with rodents in a simian—e.g., small size (∼350 g) which makes them easier to handle and house, comparatively flat cortex which improves access for a variety of recording techniques, and high fertility for a primate (obligate twinners, two litters per year) (see Table S1 for anatomical properties across model mammalian animal models for vision). However, it is not obvious how human-like marmosets may be in terms of their brain and behavior. Marmosets are a New World monkey, the simian clade most distant from humans. Moreover, even within the New Worlds, marmosets and their closest relatives were originally considered primitive and squirrel-like, distinct from the “true monkeys”^16^^,^^17^^,^^18^ — in part because of the same attributes that make them an attractive animal model, namely their diminutive size and lissencephalic cortex. Thus, simian or not, the breadth of marmosets’ utility as a small animal model for neuroscience remains unclear, particularly for high-level perception and cognition.

A marmoset’s brain is approximately two orders of magnitude smaller than a human’s (8 versus 1500 g), with orders of magnitude fewer cortical neurons in a less folded corte×.^19^ These factors are thought to lead to lesser abilities in general,^20^^,^^21^^,^^22^ and have been suggested to limit the marmosets’ high-level abilities in particular.^23^^,^^24^ However, the marmoset visual system does exhibit organizational features believed to be critical for high-level visual behavior, including perhaps most notably, a large number of hierarchically organized ventral visual cortical areas.^25^^,^^26^ At the cellular level, as one ascends the visual hierarchy, marmoset cortical pyramidal neurons demonstrate increased dendritic arborization whereas molecular and genetic work has shown that marmosets have similar inhibitory neural subtypes and laminar specific genetic markers as other primates.^27^^,^^28^^,^^29^^,^^30^ Here we tested whether marmosets can achieve high performance on sophisticated visual tasks, first comparing their behavior with that of rats — a marmoset-sized rodent but with fewer cortical areas and where other aspects of visual brain organization and cellular properties are divergent.^31^^,^^32^^,^^33^ Then we compared marmoset behavior with that of macaques and humans, substantially larger-brained fellow primates but with shared ventral visual cortical organization.^7^^,^^27^^,^^28^^,^^34^ On a simple object recognition task, marmosets outperformed rodents being more invariant to identity-preserving object transformations. On a challenging invariant object recognition task that is thought to be supported by high-level visual cortical areas,^35^^,^^36^^,^^37^ we found that the marmoset’s visual recognition behavior was robust and strikingly human-like. These results suggest that despite a small brain and body size, the marmoset appears to significantly bridge the gap often encountered between other small animal models and humans for high-level vision.

## Results

### Marmosets outperformed rats on a simple object recognition task

**Figure 1:**
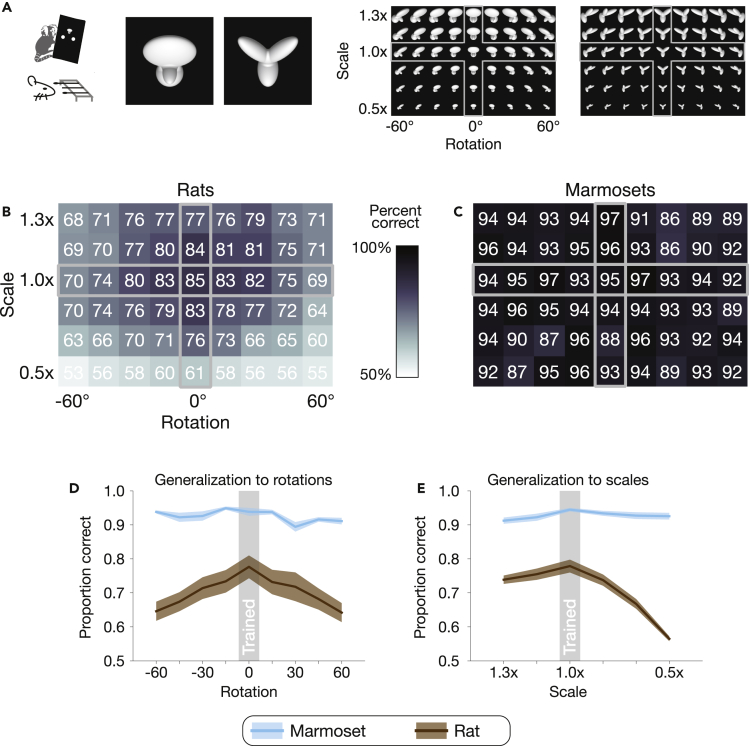
Comparing marmosets to rats, a marmoset-sized rodent (A) The two objects used in a prior rodent study^38^ (left) and all images generated by varying object rotation and scale (right). To test generalization, rats and marmosets were trained on a subset of images (the cross outlined in gray) and then evaluated on all. (B) Rat accuracy at each rotation and scale, reproduced from Zoccolan et al., 2009. Overlaid: Percent correct for each image. Gray outline indicates images on which marmosets and rats were trained. (C) Marmoset accuracy at each rotation and scale. Plotting conventions, including color scale, same as (B). (D) Generalization to novel images at each rotation. Both species were trained at 0° (frontal view). Error bars are SEM over scales. (E) Generalization to novel images at each scale. Both species were trained at 1.0x. Error bars are SEM over rotations.

We began by comparing marmoset visual behavior to that of rats, a marmoset-sized rodent that also has a relatively small brain compared to macaques and humans. Rats and other rodents (e.g., mice) have become an increasingly popular choice for studying vision in the field of neuroscience.^38^^,^^39^^,^^40^ We tested marmosets on a visual task that had been first used in rats, the results of which had been interpreted as evidence of the rodent’s sophisticated visual abilities.^38^ Animals were trained on a two-alternative forced-choice task to recognize two synthetic objects in isolation on black backgrounds and then tested on novel images of those same isolated objects under previously unencountered conjunctions of rotations and scales (Figure 1A). Using identical images and task design, we found that marmosets performed substantially better than rats (mean accuracy marmosets: 93%, rats: 71%; two-tailed t-test: t_53_ = 18.4, p = 1.14 x 10^−24^; Figures 1B and 1C). We then examined performance at a finer scale, separating out those images that marmosets and rats had encountered in training and those that they had not. When encountering novel rotations and scales, rats performed far worse, whereas the marmosets were unaffected – their recognition behavior was simply invariant (Figures 1D and 1E; ANOVA: species-by-scale interaction F_5,78_ = 39.9, p = 3.81 x 10^−20^, species-by-rotation interaction F_8,78_ = 7.62, p = 1.78 x 10^−7^). These differences were observed even though image size was scaled up to 40° for the rats, to accommodate their coarse visual acuity (visual acuity of human, marmoset, and rat, respectively: 60, 30, and 1 cycles per degree^41^^,^^42^^,^^43^).

Behavioral lapses rather than visual sensitivity may account for some of the overall performance gap between marmosets and rats. Although we do not directly measure lapse rate which is defined by asymptoting performance in psychometric functions for sensitivity,^44^ general lapses can be approximated by performance to the well-trained token image where there is presumably the least amount of visual recognition ambiguity in our task. Here, we found that marmosets had a relatively low lapse, performing at 95% for the token frontal view and size whereas rats only performed at 85% (0^o^, 1.0x images in Figures 1A and 1B). However, across even modest changes in rotation and scale, rodent performance differed far more dramatically from that of the marmosets, demonstrating clear differences in visual ability. Indeed, the performance gap between rats and marmosets reached up to 40% at more extreme rotations and scales compared to just 10% performance gap for the easiest token image (Figure 1B versus 1C). Given that absolute performance in a given species can be affected by general factors such as arousal and motivation, we focused on the relative pattern of performance across images as a specific signature of sensory processing strategies. Here, the marmoset’s sensory processing ability led to nearly identical performance across views and scales such that performance does not drop much below their estimated peak performance at the token image (blue lines in Figures 1D and 1E are relatively horizontal near 90%). Rat performance, by contrast, was dramatically affected by the same image manipulations (drop-offs in brown lines in Figures 1D and 1E).

Although marmosets’ performance on this task far surpassed that of rodents’, we found in a subsequent control experiment that the task, at least from one perspective, may not be particularly high level. In computational experiments, a linear classifier trained simply on the image pixels achieved an accuracy of 97.5%, demonstrating that high task performance could be attained with retina-like representations (i.e., image pixels). By contrast, the strongest form of invariant object recognition is thought to require multiple stages of cortical processing.^26^^,^^27^^,^^42^ For example, on difficult invariant object recognition tasks, it has been reported that linear classifiers on pixel representations fail to break chance performance, and that neural responses even from intermediate stages of the cortical hierarchy, like V4, seem unlikely to support primates’ impressive visual abilities.^36^ Thus, this first task that we had used may not be a strong test of the marmosets’ high-level abilities; we next tested marmosets on a more challenging visual task.

### Marmosets achieved high performance on a challenging high-level visual task

**Figure 2:**
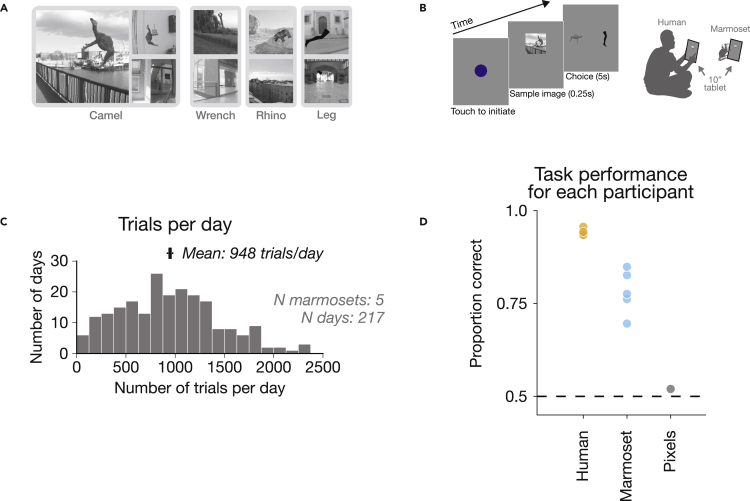
High-level invariant object recognition task and marmoset behavior (A) Example images (of 400 total) from the four objects tested. (B) Task design. (C) Histogram of trials per day by marmosets. (D) Task performance for humans, each marmoset subject, and a baseline control model. Although performance was measured on identical images, presentation time differed across marmosets and humans (250 and 50 ms, respectively).

We then evaluated marmosets’ visual behavior on a task that intuitively appears far more challenging than the task used in rodents above: the invariant identification of objects in the presence of large changes in object scale, position, and pose, superimposed on rich, naturalistic backgrounds (Figure 2A). On each trial, participants (human or marmoset) chose which of two basic-level objects (e.g., camel or wrench) was present in a briefly flashed image (Figures 2B and S1). We first tested whether this task was indeed challenging, evaluating human performance on these images (n = 7; n trials = 146,496). Consistent with previous work that used identical images,^47^ we found that humans performed this two-alternative forced-choice task well, but by no means perfectly, achieving 94% accuracy (Figure 2D). Furthermore, we found that a linear classifier trained on pixels for this task was near chance performance (50%), suggesting that this task required nontrivial visual processing.

We then tested marmosets’ abilities on this challenging high-level perceptual task. We evaluated marmosets on the same 400 images on the same touchscreen tablets as humans (n = 5 marmoset subjects; n trials = 205,667; Figure 2C and Video S1). Marmoset monkeys performed at 80% accuracy. Mean accuracy for each marmoset was 88%, 86%, 78%, 76%, and 70% (Figure 2D). We employed no subject inclusion or selection criteria—and thus these individual performances are representative of marmosets’ capabilities, rather than a reflection of a few outlier high-performing animals. The good performance of each subject demonstrates that this small-bodied, small-brained New World primate performs well at a demanding high-level perceptual task. The remaining performance gap to humans is partly because of differences in overall visual ability but may be at least partly be accounted for by differences in lapse rate given likely differences in non-sensory, cognitive factors (attention, working memory, and decision making) in the marmoset versus the human (human, n = 8: 92.4 + 2.8%; marmoset, n = 5: 79.4 + 7.5%; p = 0.0009). Therefore, we focused on the relative pattern of performance across images for a richer comparison of purely visual processing in marmosets and humans.

**Figure 3:**
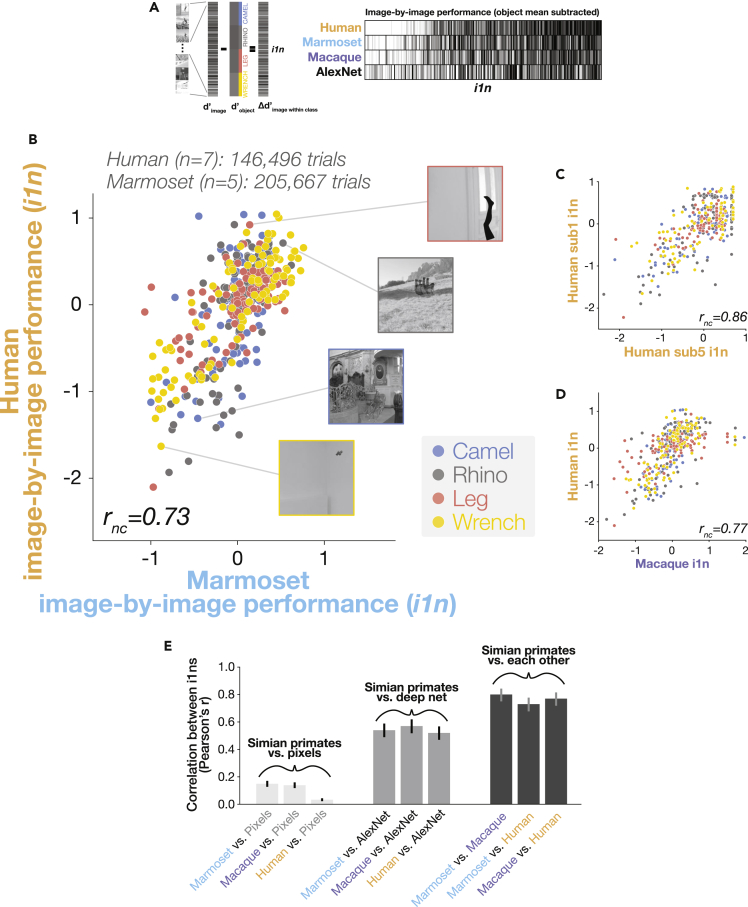
Image-by-image behavioral comparisons of simian primates (A) Image-by-image behavioral metric (*i1n*). Left: Normalization subtracts off object-level means, leaving the variance across images of that object, a fine-grained, image-by-image pattern. Right: *i1n* signatures (barcodes) for marmosets, humans, macaques, and an artificial deep neural network (AlexNet), sorted by left-out human data. (B) Scatter comparing marmoset and human behavior for each of 400 images. Color indicates object; *r*_*nc*_ denotes noise-corrected correlation (correcting for test-retest reliability of the data). Inset: Images for example points. (C) Scatter of two human participants’ against each other. (D) Scatter comparing macaque and human behavior. (E) Pairwise correlation between simian primates’ i1n performance signatures and those of pixel classifiers, a deep neural network, and each other. Error bars indicate 95% confidence intervals.

Video S1. Marmoset performing core visual object recognition task, related to Figures 2 and 3 Monkey S enters a box attached to the cage and containing a tablet plus juice tube. Monkey S is shown initiating trials then choosing one of two objects after viewing a briefly flashed sample image. Subject licks to collect reward delivered at the end of correct trials (flashed green square) and receives a timeout for incorrect trials (flashed black square).

### Marmosets exhibited human-like error patterns

Although marmosets and humans both achieved high performance on this task, it remained unclear the extent to which the two used similar strategies. Marmosets are small, arboreal primates with substantially different visual ecology and affordances than humans, and thus it is not obvious that their visual strategies would be similar to those of humans. Moreover, although the human participants in this study could be instructed as to the task design (i.e., classify objects), the marmoset monkeys had to be trained with operant conditioning over weeks to perform this task by categorizing the images (Note: They were trained with different images than used for testing). Perhaps through this use of rewards, we trained in a non-ecological behavior and, as a consequence, marmosets — while high achieving — are performing this task in a starkly different manner than the untrained humans. An alternative possibility is that, despite these differences in training, ecology, and affordances, marmosets and humans may exhibit common performance characteristics, potentially as a consequence of common aspects of visual brain architecture and neural processing.

We tested the performance signatures of marmosets and humans in this task by comparing image-by-image performance across species, under the logic that a similar visual strategy would result in similar images being easy or difficult for both marmosets and humans. We computed the difficulty of each image as a d’ then subtracting the mean difficulty of each object, leaving the variation across images for that object (Δd’_image_ = d’_image_ - d’_all_object_images_ to give object normalized image-by-image performance (*i1n*), see STAR Methods for details)(Figure 3A). Unlike one-dimensional summaries of overall performance, this image-by-image 400-length vector is a rich, high-dimensional signature of visual perception that is robust to global, non-perceptual factors like attentional lapses or motor errors. Such global factors will lead to absolute shifts in performance but leave the relative image-by-image pattern intact. This metric was therefore well-suited for comparing marmosets and humans despite their differences in mean performance.

We found that marmoset and human image-by-image performance was remarkably correlated – the two species tended to find the same images easy or difficult (r = 0.73, p = 8.91 x 10^−68^; Figure 3B). Humans have approximately twice the foveal acuity as marmosets (∼60 vs. 30 cycles per degree^41^^,^^42^), and so it was not obvious how to equate image size across the species. We therefore tested marmosets at image sizes that differed by an octave, and found that marmosets exhibited human-like behavior irrespective of size (marmosets ∼22° images, n trials = 98,378, r = 0.65, p = 2.17 x 10^−49^; marmosets ∼11° images, n trials = 107,289: r = 0.71, p = 1.35 x 10^−62^). The high correlation between marmosets and humans is rendered even more impressive by the fact that different humans exhibited slightly different image performance signatures, even after accounting for measurement noise (mean cross-subject correlation, r = 0.88; Figure 3C). These results suggest that marmosets—despite having both a brain and a body size 1/200^th^ the mass of humans’ — perform invariant visual object recognition in a strikingly human-like manner.

### Marmosets’ image-by-image object recognition behavior is nearly as human-like as that of macaques

To better frame the similarity between marmosets and humans that we measured, we also compared marmosets to rhesus macaques, as macaques are a gold standard model in visual systems neuroscience. Using previously collected macaque data on the same images,^47^ we found that marmosets and macaques exhibited highly similar image-by-image behavior (r = 0.80, p = 2.52 x 10^−90^; Figure 3D) and that, remarkably, macaques were only slightly more human-like than were marmosets (macaque-human r = 0.77; marmoset-human r = 0.73, dependent test of correlation coefficients^48^: t_397_ = 2.06, p = 0.040).

However, some details of experimental design in the previous work differed from ours (e.g., whether objects were interleaved within or across sessions; see STAR Methods for details), which raises the possibility that we may have underestimated the macaque-human similarity relative to the marmoset-human similarity, despite the use of identical images across all three species. On closer inspection, it seems unlikely that these overall task differences were particularly consequential for the image performance signatures. Human signatures collected across the two settings were highly correlated (r = 0.90, p = 1.32 x 10^−145^). Moreover, the macaque-human similarity reported in the previous work, when macaques and humans were in more similar task settings, was highly similar to what we estimated here across task settings (previous work,^47^ macaque-human r = 0.77; here, r = 0.77). The image performance signatures therefore appeared to be primarily determined by the perceptual challenge of core basic-level object recognition, which was common across experimental settings in macaques, marmosets, and humans. Put in the context of the macaque model, these results in the marmoset demonstrate how strikingly similar marmoset high-level visual behavior is to that of humans.

Finally, does this similar image-by-image performance across visual systems of two primate species reveal common processing strategies for those two systems? Or might they instead reflect something about the images — e.g., might some images simply be intrinsically more difficult than others? If the latter were the case, then any high-performing visual system would exhibit similar image-by-image performance.^49^ To test this possibility, we evaluated the performance characteristics of high-performing artificial neural networks. We found that these networks performed this task as well as marmosets but nonetheless exhibited image-by-image performance that was substantially less human-like than marmosets’ performance (Figures 3E, S3, and S4; AlexNet^50^-humans r = 0.52, dependent test of correlation coefficients t_397_ = 6.37, p = 5.29 x 10^−10^; see STAR Methods for details). This result dovetails with prior work showing a gap between models and humans,^47^ suggesting that the similarity between marmoset and human behavior is not a trivial consequence of high task performance, but instead may reflect a consequence of common principles across the brains of the two species.

## Discussion

In summary, we found that marmosets exhibited human-like core visual object recognition behavior — on the same images, marmosets were nearly as human-like as were macaques, the gold standard of visual systems neuroscience. Moreover, we found that marmosets’ visual capabilities far exceeded those of rats, a representative non-simian small animal model in visual neuroscience.^31^ Thus, aspects of core high-level perception appear to be shared across simian primates, and marmosets may offer a powerful platform for visual systems neuroscience that combines methodological advantages traditionally associated with the small, flat rodent cortex (Table S1) with high-level perceptual capabilities conventionally considered to be the exclusive purview of larger primates.

### Results in the context of previous work

It was not obvious that marmosets would exhibit such strikingly human-like higher-level visual behaviors. The extent to which marmosets are capable of higher-level perception and cognition at all has been doubted for a variety of reasons, including their relatively small brain.^23^^,^^24^ Recent work has shown similarities between marmosets and larger primates for relatively simple behaviors, such as eye movements.^6^^,^^51^ Prior work had offered initial evidence of marmoset visual recognition abilities,^6^^,^^51^^,^^52^^,^^53^^,^^54^ but that series of studies on reversal learning was not geared at characterizing visual behavior in detail as it simply involved associating a few unambiguously presented objects with reward. Here, we carefully studied how objects are recognized invariantly across a variety of transformations and in cluttered backgrounds. We were able to make tight, quantitative comparisons between species by precisely measuring their pattern of visual behavior. Our work thus uniquely adds to our understanding of marmoset behavior by demonstrating that marmosets’ image-by-image visual object recognition behavior is not only good but remarkably human-like.

### Marmoset evolution and brain organization

Over the course of evolution, species tend to get larger over time including in the primate lineage.^55^ Marmosets, however, are evolutionarily unusual as they have shrunken from far larger ancestors.^56^^,^^57^^,^^58^ This “phyletic dwarfism” has been found to lead to atypical configurations of phenotypes.^57^^,^^58^^,^^59^ Whether a consequence of this unusual evolutionary history or not, the marmoset brain has many cortical areas (∼120) by most recent accounts^60^^,^^61^^,^^62^ — for comparison, mice, macaques, and humans had ∼40, ∼140 and ∼180 areas, respectively.^62^ Thus, the marmoset monkey has a small brain size yet retains properties observed in other monkeys and humans including some anatomical separation of motion from form processing and an elaborated set of hierarchically organized visual cortical areas for form processing, accompanied by increasing dendritic arborization of pyramidal cells across this hierarchy from V1 through the prefrontal cortex,^7^^,^^28^^,^^34^^,^^63^^,^^64^^,^^65^ properties that are less prominent or mostly absent in rodents.^32^^,^^66^ Understanding how the detailed list of anatomical properties, from the cellular to the network level, leads to increased behavioral ability across the evolutionary tree is a fascinating comparative question. If we are to consolidate anatomical gains functionally, such a research effort can benefit not only from behavioral comparisons but from neural experiments followed by end-to-end modeling work integrating across levels.

### High-level vision as a test domain for cortically dependent processing

One potential concern with using a visual task to compare simian primates to non-simians (e.g., rats) is that simian primates have peripheral adaptations that increase the fidelity of the visual sensors — for example, a high-acuity fovea with minimal retinal summation.^41^ However, improved peripheral processing alone is insufficient to support the high-level visual behaviors used in our study. The neural basis of these behaviors is comparatively well established: many stages of cortical processing are required to achieve high performance on this task,^36^^,^^45^^,^^67^ and such performance cannot be supported by even complex mid-level cortical areas (like V4).^36^^,^^68^ Thus, increased sensory precision in the peripheral nervous system is, by itself, insufficient to account for marmosets’ high object recognition performance, which depends on central cortical processing. Future visual tasks can compare species differences in other visual domains that may benefit from the peripheral and central nervous system specializations found in simian vision, for example, 3D stereoscopic depth perception, color vision, and saccadic eye movements to parts of the visual scene. Here, we focused on core object recognition – discerning visual shape at a glance in 2D grayscale images – without use of color, stereo, or eye movements.

### Comparing fewer species with greater precision

Previous tests of the relationship between brain size and behavioral capabilities often summarized the behavior of an animal with a single number (e.g., mean performance on a given task).^69^^,^^70^^,^^71^^,^^72^ We take a different approach: narrower in that we only examine four species, but deeper in that we quantify the behavioral phenotype in great detail—e.g., using a 400-dimensional behavioral signature to compare marmosets, humans, and macaques. Such higher dimensional measures avoid reducing the complexities of an animal’s ethology to a single number, and thus better capture the richness of the behavioral phenotype. Richer metrics enable quantitative rigor without sacrificing holism or nuance. The cost of such higher-dimensional comparisons is that reliably measuring these signatures required hundreds of thousands of trials, which made it practical only to compare a handful of species — and encouraged the selection of species that offer a natural evolutionary experiment, like the marmoset, a phyletic dwarf within the simian primate clade. Future comparative behavioral phenotyping could further clarify the role of brain differences in producing different high-level visual abilities by using the same stimuli and tasks to assess diurnal species that are similarly small in size to the marmoset, such as lemurs or tree shrews, as well as testing non-primates like cats. In parallel, neural studies could also marry such higher-dimensional behavior phenotyping with higher-dimensional neural phenotyping to establish more nuanced relationships between brain mechanisms and behavior.

### Limitations of the study

One limitation of this study is that animals have to be trained to report object identity whereas humans generally require little training on the task logic. The effects of extensive operant conditioning in animal behavior are important to consider in contextualizing any comparison of an animal model to humans. At the same time, animal training allows collection of a large number of trials, as incentivized by reward, to perform precision psychophysics and generate large-scale datasets.

Another limitation is that we do not obtain good distributional estimates of visual object recognition behavior within a species for comparing within versus across species variance. To do so, would require assessing subject variability across a large animal cohort. However, this is practically challenging to do given limitations of resources (marmoset colony size) and experimenter time. The degree of variability across many marmoset subjects is an open question and may be more or less pronounced for sensory tasks like visual object recognition versus other complex cognitive tasks. This is an open research topic. Finally, our system of measurement using touchscreen tablets in the home environment allowed monkey and human subjects great latitude in body posture, viewing distance, and attentional state, so variability of our measures may reflect this to some degree.

## STAR★Methods

### Key resources table

**Table:** 

REAGENT or RESOURCE	SOURCE	IDENTIFIER
**Deposited data**		

Raw behavioral data for all analyses and figures	This paper and DiCarlo Lab at MIT	https://github.com/issalab/kell-et-al-marmoset-benchmarking

**Software and algorithms**		

Behavioral Experiment Software: MkTurk (mkturk.com)	Issa Lab	https://github.com/issalab/mkturk
Python with numpy, scipy, and sklearn libraries	Python Software Foundation	https://www.python.org

### Resource availability

#### Lead contact

Elias Issa, Columbia University in New York, elias.issa@columbia.edu.

#### Materials availability

This study did not generate any new unique reagents.

### Experimental model and subject details

#### Subjects

Five adult common marmosets (*Callithrix jacchus*; four male and one female) and seven adult humans (four female and three male; 22 to 42 years old) participated in our experiments. The five authors were among the human participants. The human data were collected in accordance with the Institutional Review Board of Columbia University Medical Center and gave informed consent, and the marmoset data were collected in accordance with the NIH guidelines and approved by the Columbia University Institutional Animal Care and Use Committee (IACUC). We also used data from two previously published studies from five macaques, 1,481 humans, and six rats.^38^^,^^47^ These data were collected in accordance with NIH guidelines, the Massachusetts Institute of Technology Committee on Animal Care, The Massachusetts Institute of Technology Committee on the Use of Humans as Experimental Subjects (COUHES), and the Harvard Institutional Animal Care and Use Committee.

### Method details

#### Two-way stimulus-response task

Marmoset, human, and macaque performance was measured on the identical set of images (same object, pose, position, scale, and background). See Figure S1 for these 400 images. Humans, macaques, and marmosets initiated each trial by touching a dot at the center of the screen. A sample image was then flashed (for marmosets 250 msec and for humans 50 msec; we varied presentation duration to induce a sufficient number of errors to yield reliable human image-by-image performance scores; the macaque data that was collected in previous work presented images for 100 msec). After the image disappeared, two example token images were presented (i.e., of the object on a gray background at a canonical viewing angle), and the subject had to touch which of two images was in the preceding sample image. We used a two-alternative forced-choice stimulus-response paradigm—for a given task session, whether for marmoset or human, only a pair of objects was tested (e.g., one day would be camel-wrench, another would be wrench-leg). This was varied across sessions so that images of a given object were tested in all possible two-way task settings (e.g., camel vs. rhino, camel vs. wrench, and camel vs. leg). For a given behavioral session, each object was consistently on the same side of the decision screen (e.g., camel would always be on left and wrench always on right). One motivation for using this kind of two-way stimulus-response design is that it reduces the working memory demands on the participant. As soon as the participant recognizes what is in the image, they can plan their motor action. In part because of this simplicity, the two-way stimulus response paradigm is broadly used across the neurosciences, including in rodents,^38^ and it therefore allows behavioral benchmarking for comparative studies across an array of candidate model species. Moreover, as reported in the main text, we found that the pattern of image-by-image errors was highly similar across humans who performed the two-way stimulus-response design and those that performed the match-to-sample design (the latter of which was collected in the previous work), in which pair-wise image discriminations were interleaved within a session rather than across sessions (r = 0.90; this fully-interleaved match-to-sample design was used in the previous work with the macaques). The similarity of these signatures across experimental designs suggests that when comparing model systems on purely perceptual grounds, a simplified, two-alternative stimulus-response task can serve as a universal method for comparative studies across animals—even those where a challenging match-to-sample task design may be difficult to employ.

For the rodent task, the design was similar to that of the core recognition task, but sample stimuli were not flashed and were instead present on the screen until the subject made their choice. We used this design because it mimicked Zoccolan and colleagues’ study.^38^ Instead of requiring the marmosets to use a nose-poke into two ports, we had the marmosets touch one of two white circles on-screen, one on the left and one on the right, to indicate their choice. We used white circles rather than token images to avoid allowing the marmosets to employ a pixel-matching strategy with the image on the screen.

#### Stimuli

For the core object recognition task in marmosets, we used a subset of images from a previous study in macaques (see Figure S1 for all 400 images). We briefly describe the image generation process below, but see Rajalingham et al., 2018 for more details.^47^ These stimuli were designed to examine basic-level,^73^ core object recognition. They were synthetically generated, naturalistic images of four objects (camel, wrench, rhino, and leg) displayed on a randomly chosen natural image background. The four objects were a randomly selected subset of the twenty-four used in the previous work. The spatial (x,y) position of the center of the object, the two angles parameterizing its pose (i.e., 3d rotation), and the viewing distance (i.e., scale) were randomly selected for each image. Stimuli were designed to have a high degree of variation, with disparate viewing parameters and randomized natural image backgrounds, in an effort to capture key aspects of the challenge of invariant object recognition, and to remove potential low level confounds that may enable a simpler visual system, either biological or artificial, to achieve high performance.^37^

In addition to this main set of 400 test images (100 for each object), we employed four additional sets of images in training the marmosets to do the task (for training details, see “marmoset training procedure” below). The first consisted of a single token image: the object rendered at the center of a gray background in a relatively canonical pose (e.g., side view of an upright camel). The second set consisted of 400 images of each object at random poses, positions, and scales, all on a uniform, gray background. The third set consisted of a different sample of images that were drawn from the same distribution of generative parameters as our 400 test images (i.e., variation in pose, position, and scale on randomly selected natural backgrounds). To assess the generality of our image-by-image performance scores, we collected marmoset behavior to the test images at two different sizes. Marmoset position and distance from the viewing screen was not strictly controlled but was nonetheless relatively stereotyped (see “homecage testing boxes” below), and the two image sizes subtended ∼11° and ∼22° of visual angle. For humans, we collected a single image size. Humans were not constrained in how they held the tablets on which the images were displayed, and this image size subtended ∼4-12° of visual angle, depending on how the tablet was held.

For comparing marmosets to rodents, identical images were used as in a prior study in rats; see Figure 1A for all images, and see Zoccolan et al., 2009 for more details.^38^ In brief: these stimuli were of one of two synthetic, artificial objects, which were rendered at the center of the screen on a uniform black background. Objects were rendered at one of nine different azimuthal rotations (−60° to +60°, in steps of 15°) and one of six different scales, spanning ∼1.4 octaves of size (from 0.5x to 1.3x the size of a template; for marmosets, 1.0x subtended ∼11 degrees visual angle). Rotations and scales were fully crossed for each object to yield a total of 108 images (9 rotations x 6 scales x 2 objects).

#### Web-based, homecage behavioral training system

In part inspired by the high-throughput behavioral systems used in some rodent work,^74^^,^^75^ we developed a system where behavior would be collected in parallel from a large number of animals.

##### Web-based behavioral platform

We developed the MkTurk web-based platform (*mkturk.com*) to collect the data on mobile devices (i.e., tablets) that could be deployed anywhere. We opted for a web-based system for a variety of reasons. First, MkTurk just needs a web browser to run, and as a result the setup and installation across tablets is relatively low cost, both in terms of the researcher’s time as well as money, as consumer touchscreen tablets are often cheaper than more traditional behavioral rigs. Second, such a system made it relatively turnkey to evaluate humans and marmosets in as similar environment as possible—we simply distributed the same tablets to humans, who performed the touchscreen tasks just as marmosets did. Third, being based on the web naturally enables real-time streaming of the animals’ performance to automatic analysis pipelines in the cloud, which allows seamless monitoring of the animals on any device that can access that web (e.g., a researcher’s smartphone). As a result, monitoring animal performance and troubleshooting issues in real-time is more straightforward. Moreover, since task parameters are passed from the cloud to the tablets in real time, task parameters can be adjusted on the fly remotely, which was particularly helpful during training of many subjects in parallel.

For external hardware, we leveraged the open-source Arduino platform (Arduino Leonardo) coupled to a low-power piezoelectric diaphragm pump for fluid delivery (Takasago Fluidic Systems) and RFID reader for individual animal identification (ID Innovations), all powered by a single 5V USB battery pack. Thus, our system may not be as powerful as some traditional, fully-equipped experimental rigs, but our behavior box was highly optimized for SWaP-C (size: ∼1 ft^3^, weight: ∼10 lbs, power: ∼5W, and cost: < $1,000).

##### Marmoset homecage behavior collection

Marmosets were tested in an 8” x 9” x 11” (width x height x depth) modified nest box that was attached to their housing unit. Using a standard fluid regulation paradigm, they were granted access to this box three hours a day to collect their fluid reward via the task. Marmosets could move freely in and out of the testing box for *ad libitum* access to food during the three hours, and in some instances where RFID was employed for automated identification, marmosets could engage with cagemates in social behavior when not performing the task. Marmosets performed trials on touchscreen tablets (Google Pixel C) inserted into a vertical slot in the rear of the nest box. Once in the nest box, marmoset body and head position were not strictly controlled (e.g., via chairing or head-fixing), but marmosets were encouraged into a relatively stereotyped position in a few ways. First, the boxes were augmented with dividers on both the left and right sides to restrict degrees of lateral freedom while marmosets were performing the task. Second, access to the touch screen was only available through a small (3.5” by 1.5”) armhole in the front plexiglass barrier. Third, a metal reward tube was embedded in the front plexiglass barrier and positioned at the center of the screen. Given that marmosets tended to perform well, they received regular rewards of diluted sweetened condensed milk via this tube (1 part milk: 7 parts water), and they tended to position themselves at this tube consistently, yielding a relatively stereotyped viewing position (e.g., see Video S1). We also found that marmoset weights were overall stable over the two years of the study when using fluid regulation to motivate behavioral task performance (Figure S2). By collecting behavior from animals in their housing unit in the colony, we were able to acquire data from all marmosets simultaneously. Indeed, in part because of this high-throughput behavioral system, we were able to measure performance for each of 400 images with high precision by collecting many trials per image (trial split-half correlation for marmoset’s i1n, Pearson’s r = 0.94).

##### Human behavior collection

Human data was collected on the same tablet hardware using the same MkTurk web app software. As with the marmosets, humans were allowed to perform trials in their home environments. Moreover, we also measured human image-by-image performance with high precision and reliability (trial split-half correlation for human i1n, Pearson’s r = 0.91).

#### Marmoset training procedure

Marmosets were trained on the task through a series of stages. The first stage aimed to familiarize the marmosets with the motor mapping. In this case the sample image that was flashed was simply one of the token images, and thus was the same as the images used as buttons in the decision stage. This task therefore required no visual processing beyond pixel-matching, but was helpful to familiarize marmosets with the task paradigm. In the second stage, we introduced random position, pose and scale, but still presented the images on plain gray backgrounds, and this task served as a helpful bridge between the simple motor-mapping component and the full-scale core object recognition task that was the goal of our training. In the third and final training stage, marmosets were trained on the high-variation images with disparities in position, pose, and scale and with natural-image backgrounds. When marmosets saturated performance on this final stage, we switched them to test images, and we report the behavior from these separate test images not used in the training stages.

### Quantification and statistical analysis

#### Behavioral metric: i1n

##### Definition of i1n

To compare human and marmoset core object recognition, we employed an image-by-image metric, the i1n, which has been shown in previous work to be highly discerning between visual systems^47^ and a useful tool for selecting behaviorally challenging stimuli for studying neural processing in ventral visual cortex.^76^ The i1n measures the discriminability of each image, and is designed to be a metric of a system’s sensitivity, as formalized by signal detection theory. For each image *j*, the difficulty *V*_*j*_is defined as:

$$V_{j}=z\left({HR}_{j}\right)-z\left({FAR}_{j}\right)\text{,}$$

where *V* is a 400-length vector. *HR*_*j*_ and *FAR*_*j*_ are, respectively, the hit rate and the false alarm rate—the proportion of trials that image *j* was correctly classified and the proportion of trials that any image was incorrectly classified as that object. *z( )* is the inverse cumulative density function of a Gaussian distribution, which allows evaluating the difference between the hit rate and false alarm in z-scored units. For images where a system was correct on every trial, the z-transformed hit rate is infinity, and we capped these values to be 4, as this value was just outside the ceiling of our empirically measured hit rates given the number of trials that were in each bin. No images for pooled marmosets nor humans reached this threshold; for individual human subjects (Figure 3C), sub5 had 66 of 400 points at ceiling, sub0 had 8 of 400; excluding these ceiling points did not substantially alter the correlation between the two (all points: *r*_*nc*_ = 0.861; excluding ceiling points: *r*_*nc*_ = 0.871). Nine of the four hundred macaque images reached this threshold. We then subtracted off the mean value for each object to yield the i1n. Normalizing these values but subtracting off object-level performance (the “n” in the “i1n”), makes this metric robust to mean shifts in performance for each object and helps remove differential performance across behavior sessions. In the stimulus-responses design that we employed, object correlates with session. By contrast, when trials of different pairwise discriminations are interleaved within a session like in a match-to-sample design, any day-by-day variability in performance is not correlated with object-level performance. In practice, whether or not this object-level normalization was included did not affect the main results—Figure S4 shows that key results are essentially the same when using an “i1” instead of an i1n.

##### Contextualizing the i1n

While the calculation of the metric was identical in our work and in the work by Rajalingham and colleagues,^47^ differences in the inputs to the i1ns may lead to some mild differences between the nature of each of our i1ns. First, our i1n was computed with more images. Rajalingham and colleagues reported human-macaque correlations of image-level metrics on a subset of 240 of the 2400 images tested (10 of 100 images of each of the 24 objects), because human data was somewhat expensive to acquire and, image-level metrics require large amounts of data per image to be reliably estimated. In our work, because we concentrated data collection on four objects, we collected many trials on 100 images of each object, and thus computed i1n from 400 images total. The consequences of different numbers of images are likely not particularly substantial, though high correlation coefficients are somewhat less likely between 400-dimensional than with 240-dimensional vectors (e.g., the standard deviation of a null distribution of correlations coefficients between random Gaussian 240- and 400-dimensional vectors are, respectively, 0.065 and 0.050). A second, and potentially more consequential, difference between our i1n and the i1n used by Rajalingham and colleagues is that they measured the performance of each image against twenty-three different distractor objects, whereas we only measured ours against three. By averaging over far more distractor objects, their i1n likely minimizes the effect of the choice of distractor much more than our i1n does. Our i1n therefore likely measures something between Rajalingham and colleague’s i1n and their i2n, which averages over no distractors. Nonetheless, these differences between our i1n and the i1n used in previous work may not lead to substantial differences: as reported in the main text, we find that our 400-dimensional macaque i1n (averaged over three distractors) and their 240-dimensional macaque i1n (averaged over 23 distractors) are both equally similar to the corresponding human i1ns collected in comparable situations (*r*_*nc*_(macaque,human) = 0.77 in both cases).

##### Comparing i1ns: Correcting correlation coefficients for test-retest reliability

To assess the similarity of different visual systems, we measured the consistency of the image-by-image performance of each by correlating i1ns (Pearson’s correlation coefficient). We subjected the “raw” correlation coefficients to a correction that accounts for the test-retest reliability of the data, as different systems’ i1ns will have different test-retest reliability due to, for instance, different amounts of data and/or different rates of errors. Reliability correction addresses potentially undesirable properties of comparing raw, uncorrected coefficients across pairs of systems. For instance, the consistency of a system with itself should be at ceiling (i.e., a correlation coefficient of 1), but an uncorrected correlation coefficient will have a value less than one, as it will be determined by the test-retest reliability. Because of this, the ceiling would in general be different across pairs of systems, and left uncorrected, this could lead to inaccurate inferences—e.g., one could naively conclude that system 1 and system 2 are more similar than system 1 and system 3, just because the researchers measured more reliable i1ns in system 2 than in system 3. To address these concerns, we measured and corrected for the test-retest reliability of the i1n for each system, by applying the correction for attenuation,^77^which estimates the noiseless correlation coefficient between the two—i.e., the correlation coefficient that would be observed as the number of trials goes to infinity. In doing so, we ensured that all comparisons between pairs of systems were on the same scale—i.e., the ceiling for each was indeed a correlation coefficient of 1—and thus were free to compare i1ns across these different systems.

We measured the noise-corrected correlation for a pair of systems’ i1ns by randomly partitioning trials for each image into two halves, computing i1ns for each half for each system, taking the mean of the correlation coefficients of i1ns across systems across the two split halves, and dividing it by the geometric mean of the reliability across systems (this denominator being the correction for attenuation):

$$R_{nc}=\frac{\frac{1}{2}\left(r\left(V_{a0},V_{b1}\right)+r\left(V_{a1},V_{b0}\right)\right)}{\sqrt{r\left(V_{a0},V_{a1}\right)+r\left(V_{b0},V_{b1}\right)}}$$

where *R*_*nc*_ denotes the noise-corrected correlation coefficient, *r( )* is a function that returns the Pearson correlation coefficient between its two arguments, *V*_*a0*_ and *V*_*a1*_ denote splits of trials for system a, and *V*_*b0*_ and *V*_*b1*_ denote splits of trials for system b. For each comparison, to mitigate variation due to how the data was randomly partitioned, we took the mean of this noise-corrected correlation coefficient across 1000 random partitions.

To test whether the correlation coefficients between two pairs of systems were different (e.g., marmoset-human correlation versus macaque-human correlation), we employed a dependent test of correlation coefficients,^48^^,^^78^which takes into account the dependency of the two correlation coefficients as they share a common variable (in this case humans). Accounting for this dependence increases the statistical power of the test.

Data analysis was conducted in Python and made use of the numpy,^79^ scipy,^80^ and sklearn^81^ libraries.

#### Classifiers: Deep neural networks and pixels

##### Classifiers overview

To contextualize the behavior of simian primates, we also evaluated the overall performance and image-by-image performance for a variety of artificial systems. We trained linear classifiers for our task on top of representations from deep neural networks, which have been shown to be powerful models of visual behavior and neurophysiology.^46^^,^^47^^,^^82^ We evaluated standard ImageNet-trained deep networks, downloading pretrained models from the torchvision package of PyTorch. As a low-level control, we also compared performance of linear classifiers trained on image pixels (256 x 256 pixel images), which in part assesses the extent to which features like luminance and contrast covary with labels and thus can be used to perform the task.

##### Classifiers for task performance

We evaluated linear classifiers on the penultimate layer of deep networks, training linear support vector machines (SVMs) on the same images and same binary tasks that the primates performed. We used a hinge loss and L2 regularization. The features were nonnegative, because they were the activations of rectified linear units in the network, and so we did not center the data as that would alter the consequences of L2 regularization We instead experimented with allowing the model to learn an intercept term or not, and observed similar results in both cases; we report results when learning an intercept. To select the strength of regularization, we searched over a range of 21 logarithmically spaced hyperparameters and selected hyperparameter values via 80-20 splits within the training set. To mitigate variation of results due towhich images were selected for training or testing, we trained 1000 classifiers on random train-test partitions.

We confirmed that we were saturating performance with the amount of training data, by varying the number of images we trained on, using values of 10, 50, 90, and 99 images per object (out of 100 total images per object). Moreover, in a pilot experiment we established that we were not underestimating deep network performance due to the fact that the marmosets, humans, and macaques were able to generate an expanded “training set” of retinal activations because they were free to fixate anywhere during image presentation. To test this possibility, we generated an augmented training set that was 100x bigger, taking 100 random crops of different sizes and center locations (but not horizontal reflections) to mimic the varied fixations that marmosets and humans were allowed. We then trained classifiers on AlexNet representations, as that was the network with the lowest performance and thus greatest potential for improvement. It seemed plausible that this augmented dataset would lead to improved performance, as convolutional networks are not invariant to modest translation or scaling,^83^ and this kind of data augmentation is a central component of contemporary deep network training pipelines. Nonetheless, expanding the dataset hardly improved decoder performance at all, demonstrating that, at least for the amount of training data used here, varied “fixations” do not substantially improve classifier performance on top of a pretrained network, and thus we appear not to be overestimating the difficulty of this task for deep network representations.

##### Classifiers for i1ns

To evaluate the i1n of artificial visual systems (deep neural networks and pixel representations), we computed the distance of each image from the hyperplane learned by a linear SVM. We took 50-50 splits of images, trained classifiers on each half and evaluated distances for the left-out images. To derive an i1n for each artificial visual system, we averaged the distance for each image over its value in the three tasks, and subtracted off mean values for each task. To get highly reliable estimates of the network’s i1n, we performed this procedure 1,000 times for each task and each network, as well as for pixel representations. The ability to run arbitrarily large number of classifiers *in silico* yields the ability to drive the split-half correlation of the resulting i1ns arbitrarily high. Indeed, while the distances from the hyperplane are relatively reliable across individual partitions (for network classifiers, correlation coefficient ranges from 0.86 to 0.93; for pixels-based classifier: 0.66), the reliability of the distances averaged across 500 random train-test partitions is greater than 0.99 for all classifiers. Because of these exceedingly high test-retest reliabilities, we did not apply noise correction to the classifier i1ns learned from pixel or deep network features.

##### Classifiers for rodent task

To evaluate the kinds of visual representations required to generalize on the task used by Zoccolan and colleagues, we mimicked the training that the animals received in how we trained our classifiers. We trained classifiers on pixels from the 28 images used initially in training (14 images at either 0° rotation or 1.0x scale for each of two objects), and evaluated the performance of the resulting classifier on the 80 held-out test images (40 images for each of the two objects). We again used a linear SVM classifier with a hinge loss and L2 regularization and selected the regularization strength hyperparameter via cross-validation within the 28 train images. We evaluated 5 candidate values for regularization strength which were logarithmically spaced, and performed 10 random splits within the training set, training classifiers with each regularization strength on 23 images and evaluating the quality of the fit with the remaining 5. We then selected the regularization strength that performed best on left-out train images, and trained a single classifier with all 28 images with this regularization coefficient. We evaluated the performance of this classifier on the unseen 80 test images, and found that it classified all but two correctly (overall performance: 97.5%)—the two that it got incorrect were at the smallest size (0.5x) combined with the most dramatic rotation (60° or −60°) (bottom left and right corners of Figure 3A). Given the already high performance of image pixels, we did not further evaluate deep network performance on this task (but see Vinken and Op deBeeck^84^).

### Additional resources

Custom software for behavioral psychophysics (MkTurk): https://github.com/issalab/mkturk.

## Data Availability

All data and code to reproduce the main results are available at the following url: https://github.com/issalab/kell-et-al-marmoset-benchmarking.
