# Supplementary material for: Marmoset core visual object recognition behavior is comparable to that of macaques and humans
Source: iScience. 2022 Dec 10;26(1):105788. doi: 10.1016/j.isci.2022.105788 (PMC9804140; doi:10.1016/j.isci.2022.105788)
Supplement: Document Figures S1–S4 and Table S1 [file mmc1.pdf]

iScience, Volume *26*

Supplemental information

Marmoset core visual object recognition behavior is comparable to that of macaques and humans

Alexander J.E. Kell, Sophie L. Bokor, You-Nah Jeon, Tahereh Toosi, and Elias B. Issa

	Non-primate			Prosimian	Simian primates		
	Mouse	Rat	Treeshrew	Mouse lemur	Marmoset	Macaque	Human
Mass (kg)	0.025	0.400	0.160	0.060	0.300	7.500	80
Gyrification index	1.02[1,2]	1.04[1,2]	1.04[2]	1.11[1]	1.18[1,2]	1.75[1]	2.56[1]
Brain weight	0.4	2	3	1.7	8	80	1500
Number of cortical areas	~40[3]	?	~40[4]	~50[5]	~120[3]	~140[3]	~180[3]
Visual acuity (cycles per degree)	0.5[6]	1[6]	2[7]	5[7]	30[7]	50[7]	60[7]
Peak retinal cone density (cones/mm²)	11,000-18,000[7]	?	36,000[7]	7,500-8,000[7]	152,000-360,000[7]	134,000 160,000[7]	100,00-324,000[7]
Eye ball size (axial diameter, mm)	5.2[8]	5.6[8]	8[8]	9.2[8]	11.3[8]	20[8]	24[8]
Diurnal or nocturnal	Nocturnal	Nocturnal	Diurnal	Nocturnal	Diurnal	Diurnal	Diurnal
Last common ancestor with humans (MYA)	>90[9]	>90[9]	>90[9]	87[9]	43[9]	32[9]	N/A

Table S1. Species comparison table, Related to Introduction and Discussion. The marmoset may offer the strengths of small animal models (small size, relatively flat brain), while preserving many desirable aspects of a larger primate model like the macaque (number of cortical areas and high acuity vision, which may stem from their evolutionary distance and visual ecology). Each column is a species, and each row a property of the animal. Arguably more desirable traits in a neuroscience animal model are shaded green, less desirable red. MYA: Million years ago; gyrification index: the ratio of total cortical surface area over exposed cortical surface area. Estimates of the number of cortical areas vary depending on the method (cytoarchitecture, connectivity, etc.); however, one generally accepted trend is that the number of areas increases substantially in the simian primate[10]. Estimates of mouse and human number of areas are considered the most accurate because of the convergence across multiple measurements[3], while the number of cortical areas in emerging models such as the tree shrew and mouse lemur are less well established (though see[4,5]).

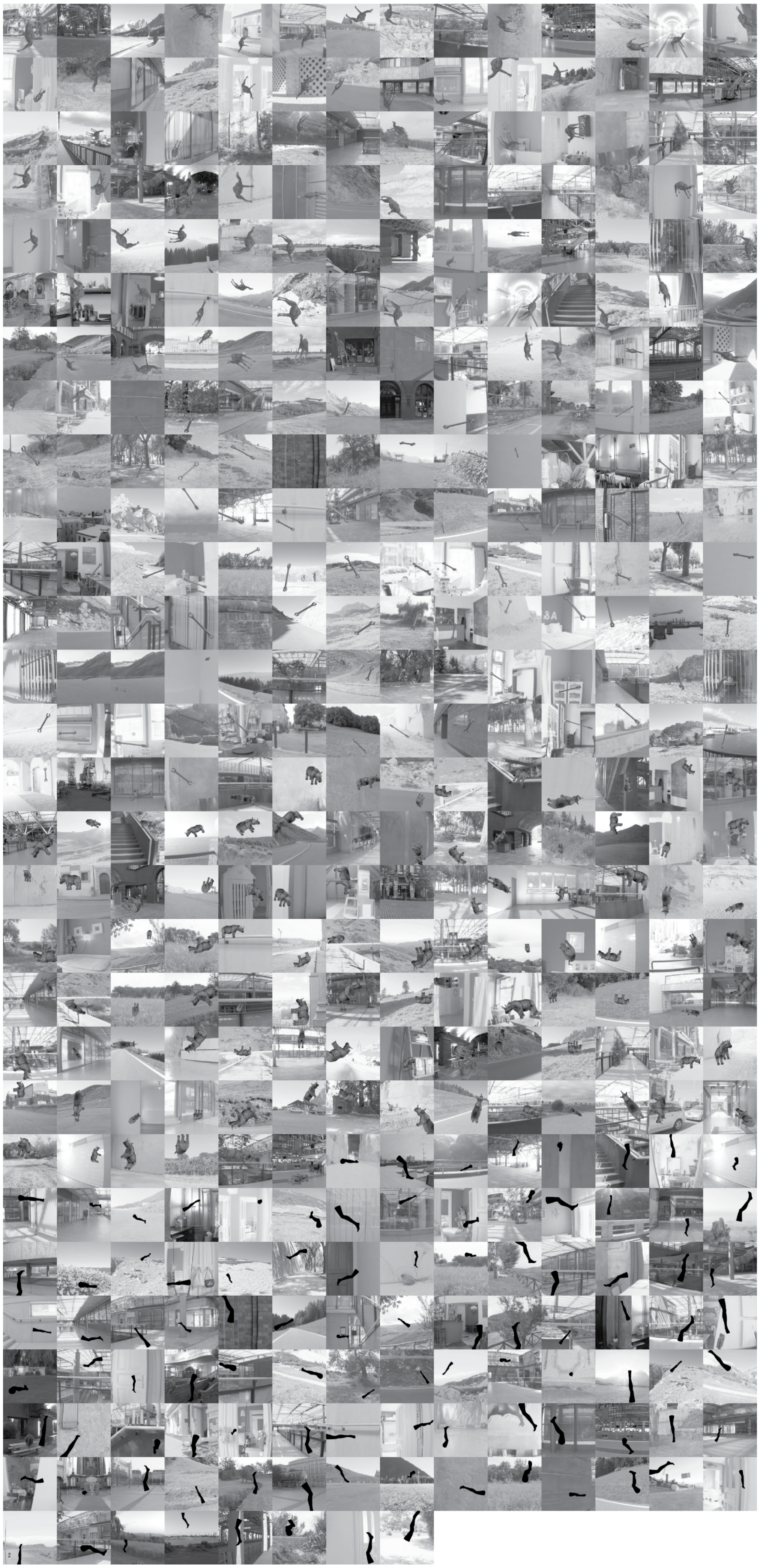

Figure S1. All 400 images, Related to Figure 1. All four hundred images of camels, wrenches, rhinos, and legs superimposed on natural backgrounds. We measured marmoset and human performance on each of these images and compared these performances with each other and with macaque performance on the same images collected in previous work[11].

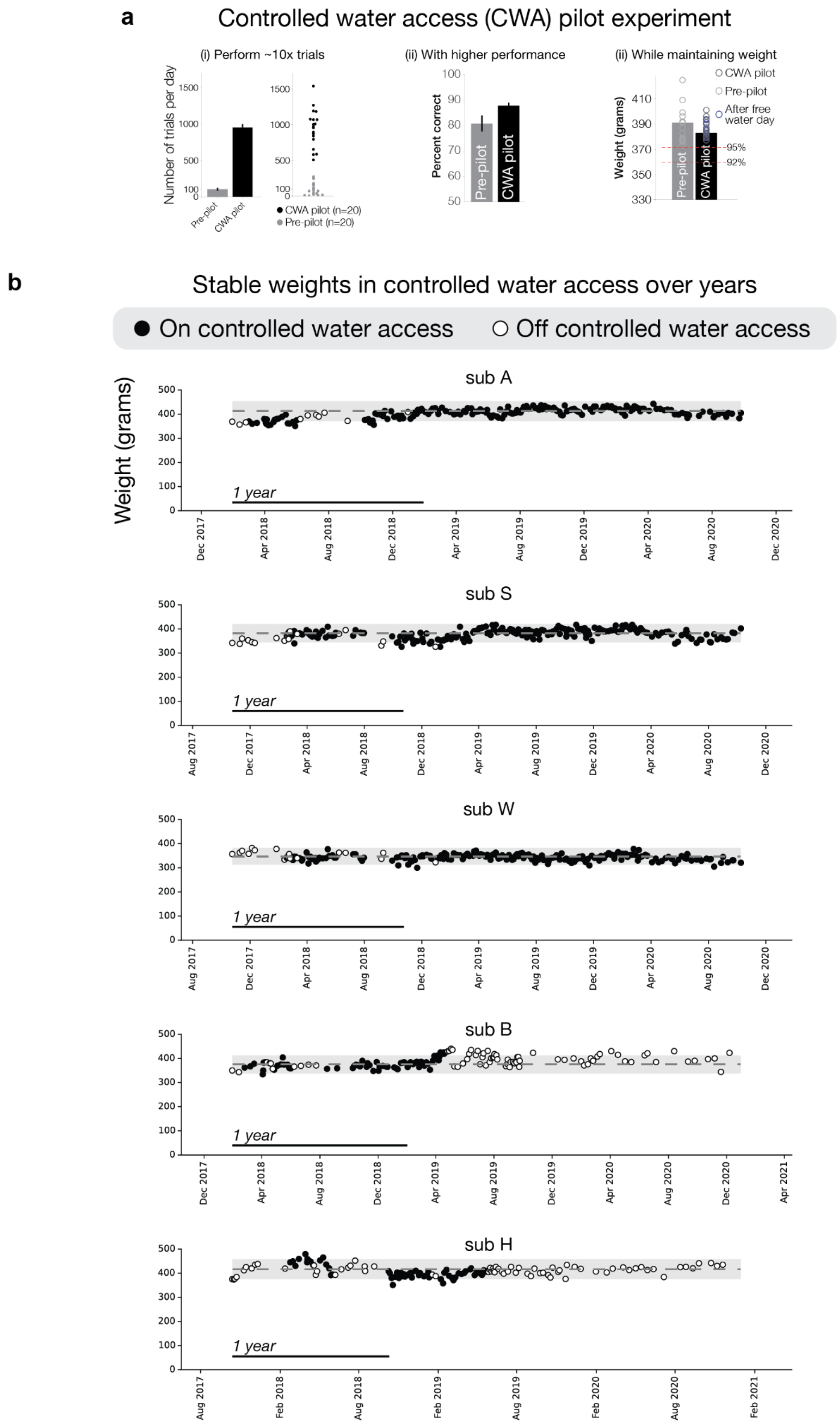

Figure S2. Controlled water access is safe and effective in marmosets over extended periods of time, Related to Methods.

a. **Controlled water access pilot experiment.** In controlled water access paradigms, animals only receive fluid from performing the task. This approach, though standard in macaque and rodent

work, had been largely avoided in marmosets, potentially due to a reputation of being too fragile. We evaluated the potential of using water regulation in marmosets, proceeding carefully at first with a small pilot experiment designed to test whether controlled water access may be safe and effective. We directly compared behavior of one animal when measured under controlled water access versus *ad libitum* water access. In an effort to give the *ad libitum* condition the greatest chance of success, in this condition we removed the marmoset's access to water three hours before the task to induce a greater probability of thirst and used a high-value reward during the task (diluted sweetened condensed milk). Nonetheless, under the controlled water access condition, the marmoset performed approximately 10x the number of trials (i), with higher performance (ii), while maintaining a relatively stable weight (iii).

b. **Stable weights in chronic controlled water access for more than a year.** In follow-up studies using more animals and longer time periods, animals maintained stable weights under controlled water access for more than a year. Dashed line indicates baseline weight measured before CWA. Shaded area is that baseline +/-10%.

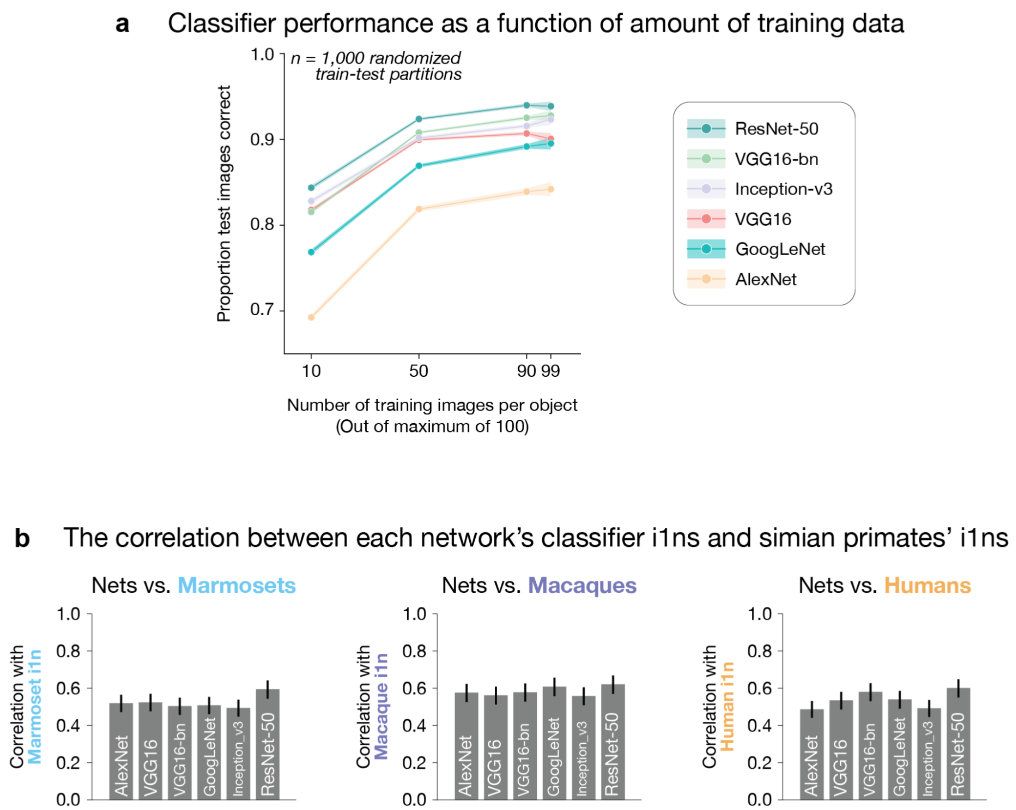

Figure S3. Deep networks: Performance as a function of amount of training data and similarity to simian primates in i1n, Related to Figures 2 and 3.

a. **Deep network performance on core recognition task.** The difficulty of a discrimination task varies drastically depending on the choice of stimuli[12], and so we verified that our images yielded a challenging task not only by assessing human performance as we report in the main text, but also by evaluating the performance of state-of-the-art engineering systems. We trained binary classifiers atop the penultimate layer of artificial deep neural networks, using 10, 50, 90, or 99 images per object (total images per object: 100), varying the training regime to establish that we reached a performance plateau with the amount of training data that we had available. Each line is a different network (VGG16-bn denotes a VGG16 architecture trained with batch normalization). Error bars are SEM over 1,000 classifiers trained on random train-test partitions; standard errors increase as amount of training data increases since the test dataset size concomitantly decreases (i.e., train size of 99 leaves only 1 image per object for testing). As we report in the main text, the raw input was insufficient to support task performance, as image pixel representations performed near the 50% chance level. Additional sensory processing, as instantiated by deep artificial neural networks, yielded performance at 84-94% indicating that high performance was achievable, but that even high-quality computer vision systems did not readily perform the task perfectly. These analyses complement the human performance results in demonstrating that recognizing the objects in these images requires nontrivial sensory computation.

b. Correlation between classifiers on different networks' features and simian primates. From left to right, we compared marmoset, macaque, and human i1ns with i1n of the six networks. The

consistency between networks and simians was relatively similar across the different networks. Error bars indicate 95% confidence intervals.

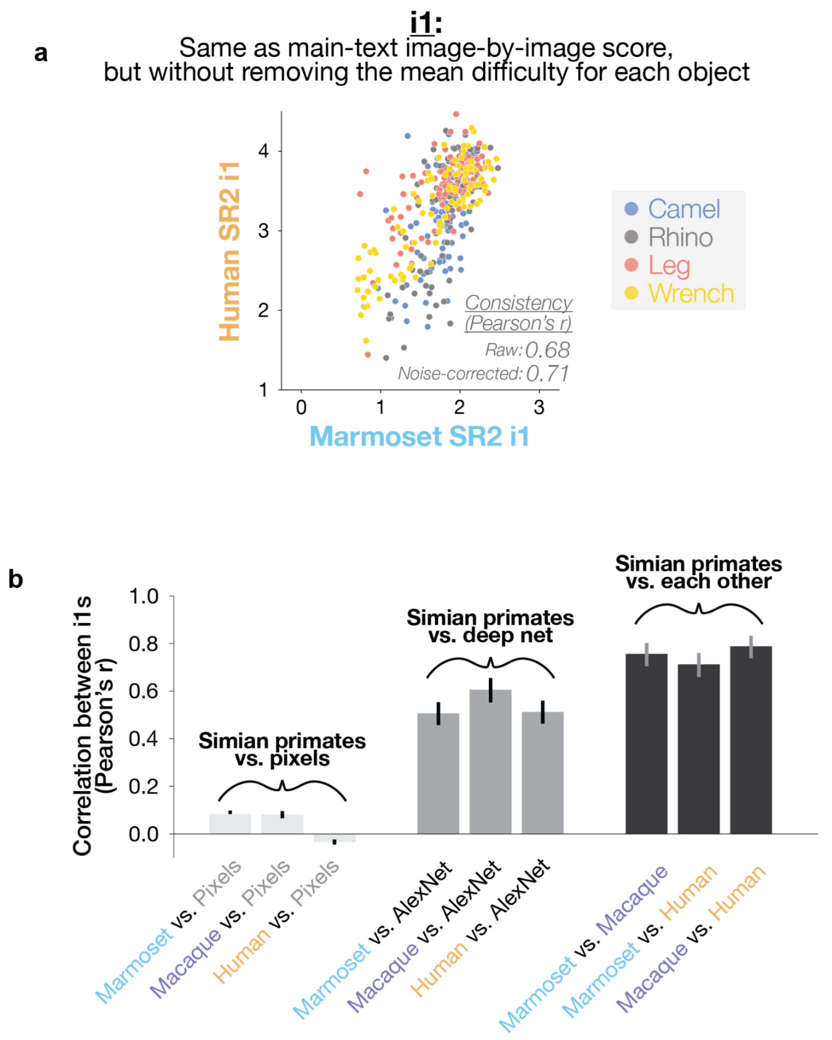

Figure S4. Image-by-image behavioral similarity between marmosets and humans is robust to details of the comparison metric, Related to Figure 3.

a. The image-by-image difficulty score in the main text (i1n) had the mean difficulty of the object removed from each image-wise score (see main text Fig. 3a). Previous work had found that such a metric of within-object image difficulty is highly discriminating between model systems (Rajalingham et al., 2018)[11]. Here, we plot the same image-by-image metric, but without this object-level de-meaning step. We find that the similarity between marmosets and humans is robust to this detail of the comparison metric. Additionally, as this metric does not include a de-meaning step, this plot shows the difference in overall performance level between marmosets and humans—marmoset image-wise d' ranges from just under 1 to approximately 2.5, while human d' scores range from just under 2 to just over 4. While overall performance varies across simians (as shown in main text Fig. 2d), the comparative difficulty of each image was relatively robust as seen in the high correlation between marmosets and humans (here and main text, Fig. 3).

b. When we compare the unnormalized i1s from pixel classifiers, deep network classifiers, and simian primates, we see a similar pattern of results as we see with i1ns (compare to main text, Fig. 3e). Error bars indicate 95% confidence intervals.

Supplemental References

1. Zilles, K., Palomero-Gallagher, N., and Amunts, K. (2013). Development of cortical folding during evolution and ontogeny. Trends Neurosci. *36*, 275–284. 10.1016/j.tins.2013.01.006.
2. Ventura-Antunes, L., Mota, B., and Herculano-Houzel, S. (2013). Different scaling of white matter volume, cortical connectivity, and gyrification across rodent and primate brains. Front. Neuroanat. *7*. 10.3389/fnana.2013.00003.
3. Essen, D.C.V., Donahue, C.J., Coalson, T.S., Kennedy, H., Hayashi, T., and Glasser, M.F. (2019). Cerebral cortical folding, parcellation, and connectivity in humans, nonhuman primates, and mice. Proc. Natl. Acad. Sci. *116*, 26173–26180. 10.1073/pnas.1902299116.
4. Wong, P., and Kaas, J.H. (2009). Architectonic Subdivisions of Neocortex in the Tree Shrew (Tupaia belangeri). Anat. Rec. *292*, 994–1027. 10.1002/ar.20916.
5. Saraf, M.P., Balaram, P., Pifferi, F., Gămănuţ, R., Kennedy, H., and Kaas, J.H. (2019). Architectonic features and relative locations of primary sensory and related areas of neocortex in mouse lemurs. J. Comp. Neurol. *527*, 625–639. 10.1002/cne.24419.
6. Prusky, G.T., West, P.W.R., and Douglas, R.M. (2000). Behavioral assessment of visual acuity in mice and rats. Vision Res. *40*, 2201–2209. 10.1016/S0042-6989(00)00081-X.
7. Kirk, E.C., and Kay, R.F. (2004). The Evolution of High Visual Acuity in the Anthropoidea. In Anthropoid Origins: New Visions Developments in Primatology: Progress and Prospects., C. F. Ross and R. F. Kay, eds. (Springer US), pp. 539–602. 10.1007/978-1-4419-8873-7_20.
8. Veilleux, C.C., and Kirk, E.C. (2014). Visual Acuity in Mammals: Effects of Eye Size and Ecology. Brain. Behav. Evol. *83*, 43–53. 10.1159/000357830.
9. Perelman, P., Johnson, W.E., Roos, C., Seuánez, H.N., Horvath, J.E., Moreira, M.A.M., Kessing, B., Pontius, J., Roelke, M., Rumpler, Y., et al. (2011). A Molecular Phylogeny of Living Primates. PLOS Genet. *7*, e1001342. 10.1371/journal.pgen.1001342.
10. Kaas, J.H. (2019). Chapter 3 - The origin and evolution of neocortex: From early mammals to modern humans. In Progress in Brain Research Evolution of the Human Brain: From Matter to Mind., M. A. Hofman, ed. (Elsevier), pp. 61–81. 10.1016/bs.pbr.2019.03.017.
11. Rajalingham, R., Issa, E., Bashivan, P., Kar, K., Schmidt, K., and DiCarlo, J. (2018). Large-scale, high-resolution comparison of the core visual object recognition behavior of humans, monkeys, and state-of-the-art deep artificial neural networks. J. Neurosci. *38*, 7255–7269.
12. Pinto, N., Cox, D.D., and DiCarlo, J.J. (2008). Why is Real-World Visual Object Recognition Hard? PLOS Comput. Biol. *4*, e27. 10.1371/journal.pcbi.0040027.
